# An Insertion Sequence-Dependent Plasmid Rearrangement in *Aeromonas salmonicida* Causes the Loss of the Type Three Secretion System

**DOI:** 10.1371/journal.pone.0033725

**Published:** 2012-03-14

**Authors:** Katherine H. Tanaka, Stéphanie Dallaire-Dufresne, Rana K. Daher, Michel Frenette, Steve J. Charette

**Affiliations:** 1 Institut de biologie intégrative et des systèmes, Pavillon Charles-Eugène-Marchand, Université Laval, Quebec City, Quebec, Canada; 2 Centre de recherche de l'Institut universitaire de cardiologie et de pneumologie de Québec, Quebec City, Quebec, Canada; 3 Département de biochimie, de microbiologie et de bio-informatique, Faculté des sciences et de génie, Université Laval, Quebec City, Quebec, Canada; 4 Groupe de Recherche en Écologie Buccale, Faculté de médecine dentaire, Université Laval, Quebec City, Quebec, Canada; University of Osnabrueck, Germany

## Abstract

*Aeromonas salmonicida*, a bacterial fish pathogen, possesses a functional Type Three Secretion System (TTSS), which is essential for its virulence. The genes for this system are mainly located in a single region of the large pAsa5 plasmid. Bacteria lose the TTSS region from this plasmid through rearrangements when grown in stressful growth conditions. The *A. salmonicida* genome is rich in insertion sequences (ISs), which are mobile DNA elements that can cause DNA rearrangements in other bacterial species. pAsa5 possesses numerous ISs. Three IS*11*s from the IS*256* family encircle the rearranged regions. To confirm that these IS*11*s are involved in pAsa5 rearrangements, 26 strains derived from strain A449 and two Canadian isolates (01-B526 and 01-B516) with a pAsa5 rearrangement were tested using a PCR approach to determine whether the rearrangements were the result of an IS*11*-dependent process. Nine out of the 26 strains had a positive PCR result, suggesting that the rearrangement in these strains were IS-dependent. The PCR analysis showed that all the rearrangements in the A449-derived strains were IS*11*-dependent process while the rearrangements in 01-B526 and 01-B516 could only be partially coupled to the action of IS*11*. Unidentified elements that affect IS-dependent rearrangements may be present in 01-B526 and 01-B516. Our results suggested that pAsa5 rearrangements involve IS*11*. This is the first study showing that ISs are involved in plasmid instability in *A. salmonicida*.

## Introduction

The Gram-negative bacterium *Aeromonas salmonicida* subsp. *salmonicida* is the etiological agent of furunculosis, a disease that causes septicemia and necrosis, especially in salmonids [1], [2]. This disease has an important economic impact on the fish farming industry.

*A. salmonicida* possesses a wide variety of virulence factors, including extracellular proteases, lipases, and a functional type three secretion system (TTSS) [3], [4]. The TTSS, a virulence system shared by many Gram-negative bacteria, can translocate effector proteins into host cells where they can disrupt cell processes and disturb immune responses [5], [6]. The TTSS is essential for the virulence of *A. salmonicida* in fish and *Dictyostelium discoideum* amoeba [7], [8], [9], [10].

While *A. salmonicida* can harbor many plasmids, plasmid occurrence varies among strains. For example, reference strain A449 carries pAsa4, a large plasmid that has not yet been clearly documented in other *A. salmonicida* strains [11]. While many *A. salmonicida* strains carry four small plasmids (pAsa1, pAsa2, pAsa3, and pAsal1), A449 lacks pAsal1 [12]. Most TTSS encoding genes are located in a single locus on pAsa5, except for the effector proteins encoding genes *aopP* located on pAsal1 and *aexT* located on the chromosome, [11], [13], [14], [15]. Some studies have shown that pAsa5 is an unstable plasmid [10], [13]. While it was first thought that pAsa5 is lost when *A. salmonicida* is grown in stressful conditions, such as at 25°C [13], it appears that pAsa5 undergoes a rearrangement that leads to the loss of TTSS and, as a result, a loss of virulence [10].

**Figure 1:**
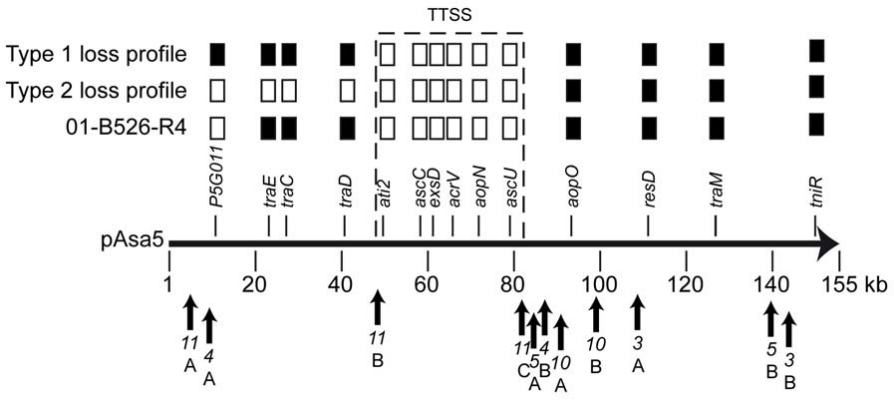
Insertion sequences (IS*11*s) are flanking the regions of the pAsa5 plasmid in *A. salmonicida* that are lost. The pAsa5 linear plasmid map shows the TTSS region and loss profiles of the various *A. salmonicida* strains from Table 1 and obtained in a previous study by growing strains at 25°C for two weeks [10]. Black rectangles indicate positive PCR signals for the corresponding genes while white rectangles indicate negative signals. Type 1 loss profile is the most common of the three [10]. Arrows below the map indicate ISs on pAsa5.

According to Daher et al., two major kinds of rearrangements occur on pAsa5 that result in different loss profiles [10]. The most frequent rearrangement leads to type 1 loss profile (Figure 1), where only the TTSS locus is lost. A less frequent rearrangement leads to type 2 loss profile where the TTSS locus is lost together with a 40-kb region located just upstream (Figure 1).

A DNA sequence analysis of pAsa5 revealed the presence of 11 copies of insertion sequences (ISs) (Figure 1) [11]. The presence of large numbers of ISs is not exclusive to pAsa5 in *A. salmonicida*. The genome of strain A449 carries 88 copies of various ISs, which is a higher number than generally encountered in γ–proteobacteria [16]. Two copies of IS*11* (also named IS*As3* [11]), a member of the IS*256* family [11], are contiguous to the TTSS-encoding region of pAsa5 (Figure 1). Members of the IS*256* family have been detected in both Gram-negative and Gram-positive eubacteria [17]. These mobile genetic elements are part of the Tn*4001* composite transposon [18]. The presence of several copies of these ISs has been associated with homologous genomic recombination [19] and replicon fusion [20]. Based on these findings, we hypothesized that the copies of IS*11* on pAsa5 (IS*11*A, IS*11*B, and IS*11*C) (Figure 1) contribute to plasmid rearrangements that lead to the loss of the TTSS. We thus investigated whether IS*11* is involved in pAsa5 rearrangements. We used a PCR approach based on primers that target IS-flanking regions to analyze a bank of TTSS-deficient strains.

## Materials and Methods

### Bacterial strains and growth conditions

The *A. salmonicida* parental and derivative strains used in this study are listed in Table 1. The derivative strains were generated in a previous work [10]. Briefly, these derivatives were produced by inoculating parental strains from frozen stock cultures on furunculosis agar (10 g of Bacto-Tryptone, 5 g of yeast extract, 1 g of L-tyrosine, 2.5 g of NaCl, and 15 g of agar per liter of distilled water) [21]. The agar plates were incubated for two weeks at 25°C. PCR genotyping was then performed to detect deletions in pAsa5 [10]. Derivatives that had lost the TTSS region (type 1 loss profile) and derivatives that had also lost the upstream region (type 2 loss profile) were used in the present study. 01-B526-R4 was kept for further analyses even though it had lost the TTSS region as well as another gene 40 kb upstream from TTSS [10]. All the strains were inoculated on furunculosis agar from frozen stock cultures and were grown at 18°C for two or three days before being lysed for genotyping.

**Table 1:** *A. salmonicida* strains used in this study and compilation of the various analyses done on these strains.

Strain	Reference	Type of loss profile as already determined [10]	Positive PCR product suggesting an A–C rearrangement^a^	Positive PCR product suggesting a B–C rearrangement^b^	Presence of pAsal1^c^
A449^d^	[8]	None	N/A^e^	N/A	-
A449-R1	[10]	1	-	+	-
A449-R2	[10]	2	+	-	-
A449-R3	[10]	1	-	+	-
A449-R4	[10]	1	-	+	-
A449-R5	[10]	2	+	-	-
01-B526^d^	[27]	None	N/A	N/A	+
01-B526-R2	[10]	1	-	+	+
01-B526-R3	[10]	1	-	-	+
01-B526-R4	[10]	Undetermined^f^	+	-	+
01-B526-R5	[10]	1	-	+	-
01-B526-R6	[10]	1	-	-	+
01-B526-R7	[10]	1	-	-	-
01-B526-R8	[10]	1	-	-	-
01-B526-R9	[10]	1	-	-	+
01-B526-R10	[10]	1	-	-	+
01-B526-R11	[10]	1	-	-	+
01-B526-R12	[10]	1	-	-	-
01-B526-R13	[10]	1	-	-	+
01-B526-R14	[10]	1	-	-	+
01-B526-R15	[10]	1	-	-	+
01-B526-R16	[10]	1	-	-	+
01-B526-R17	[10]	1	-	-	+
01-B526-R18	[10]	1	-	-	+
01-B526-R19	[10]	1	-	-	+
01-B516^d^	[10]	None	N/A	N/A	+
01-B516-3	[10]	1	-	-	+
01-B516-11	[10]	1	-	-	+
01-B516-30	[10]	1	-	+	+

a with 11AF and 11CR primers (Table 2).

b with 11B1F and 11CR primers (Table 2).

c see Figure 5.

d Parental strain.

e Not applicable.

f 01-B526-R4 had lost the genes corresponding to a type 1 loss profile as well as *P5G011*, another gene 40 kb upstream from the TTSS region (Figure 1).

### PCR analyses

DNA templates were prepared by lysing one bacterial colony of the parental and derivative strains in 20 µl of SWL buffer (50 mM KCl, 10 mM Tris, pH 8.3, 2.5 mM MgCl_2_, 0.45% NP-40, and 0.45% Tween 20) [22]. The lysates were heated at 95°C for 5 min. The PCR mixture contained 4 µl of 5× Go-Taq buffer (Promega, Madison, WI, USA), 1.6 µl of 12.5 mM dNTP, 1.3 µl of forward and reverse primers (100 ng/µl each), 0.1 µl of GoTaq (5 U, Promega), 10.7 µl of H_2_O, and 1 µl of DNA template [22]. The PCR program was as follows: 2 min 30 s at 95°C, 30 cycles of 30 s at 95°C, 30 s at 55°C, and 2 min at 68°C, followed by a final extension for 10 min at 68°C. The samples were separated on 1% agarose gels and stained with 0.5 µg/ml ethidium bromide. The PCR analyses were performed at least twice.

The PCR primers are listed in Table 2. Primers not found in the literature were designed using a primer design software available online on the Integrated DNA Technologies website (www.idtdna.com). The sequences of the chromosome and pAsa5 plasmid of *A. salmonicida* A449 and the sequence of the pAsal1 plasmid from *A. salmonicida* JF2267 were used to design the primers (GenBank accession numbers: CP000644, CP000646, and AJ508382) [11], [14].

**Table 2:** Primers used in this study.

Primer	Sequence (5′-3′)	Reference
Control primers		
*tapA* F	ACATGAAGAAGCAATCAGGC	[28]
*tapA* R	AGAGGTCATGCGTTAGCAG	[28]
*traC* F	TGCACTATCCCCAGCTATCC	[10]
*traC* R	TCGGTAATCGCGGTCTTGTC	[10]
*exsD* F	AGAAGTGATCCTGACCCAAGGCAA	[10]
*exsD* R	TTGCAACGACTGTTGCCAAGAACC	[10]
*resD* F	TCAGAAACTTGGCCATCGCTCACA	[10]
*resD* R	TGATGTGCAGATTTCCCTGGAGCA	[10]
pAsal1 F	TAACATGGGTGAGTCAGGA	[12]
pAsal1 R	TGCATGTTTGTAAAAAGTAGGTG	[12]
IS-associated primers (pAsa5)		
11AF	AATAGGTGTCGCAAGCTGGGTTGA	This study
11B1F	GCGCACCACCACCATTTAATGTCA	This study
11CR	AACTGGCAAGGATAGAGCTGCTGA	This study
IS-associated primers (chromosome)		
11D1F2	AAAGAATCGTAGCTTGCTTGCGGG	This study
11D2R2	GCCATCCGTGAATTGCCGTTTGAA	This study

PCR controls have been performed throughout the study to confirm the presence of DNA in bacterial lysates using a primer pair detecting the *tapA* gene (Table 2), which is carried by the chromosome. In addition, to ensure that lysates showing no amplification with primers targeting genes *traC*, *exsD* and *resD* found on pAsa5 was not due to inappropriate performance of the primers or the PCR assay, these primers were also tested in parallel with lysates of DNA known to contain these genes (typically lysates of A449). Finally, all new primers developed in this study have been tested in an appropriate PCR assay to confirm that they do not produce unspecific amplification alone or in combination.

### Amplicon sequencing

Positive PCR products that confirmed B–C or A–C rearrangements (i.e. 2 kb products obtained with primer pairs 11B1F-11CR and 11AF-11CR, respectively) were purified using PureLink PCR purification kits (Invitrogen, Carlsbad, CA, USA) and were sent to the genome analysis platform (IBIS, Université Laval, QC, Canada) for sequencing. A nucleotide BLAST analysis of the sequences was performed to ensure that the PCR products contained recombined ISs.

### Plasmid DNA isolation and restriction enzyme profiles

Plasmid extracts from the 01-B526- and 01-B516-derived strains were prepared using the Wizard Plus SV Minipreps DNA purification system (Promega). The extracts (5 µL) were mixed with 3.5 µl of H_2_O, 1 µl of 5× reaction buffer (New England Biolabs, Ipswich, Massachusetts, USA), and 0.5 µl of EcoRI (5U, New England Biolabs). The mixtures were incubated for 2 h at 37°C. The DNA samples were separated on 0.7% agarose gels and stained with ethidium bromide (0.5 µg/ml).

## Results and Discussion

**Figure 2:**
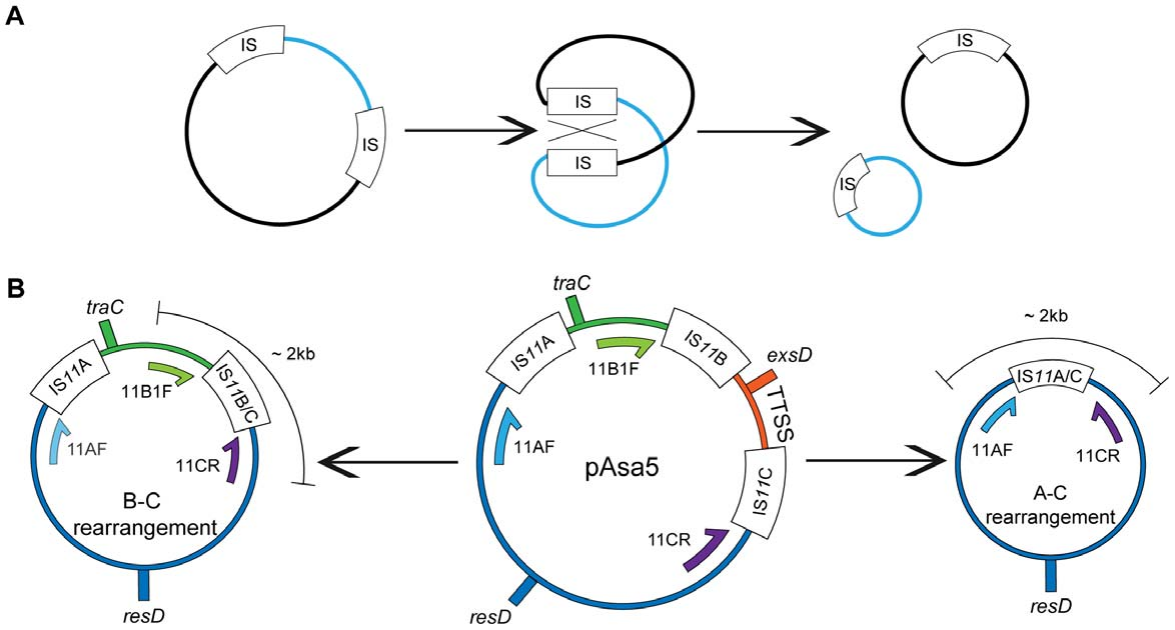
IS-dependent rearrangement may explain loss profiles 1 and 2, and can be assessed by PCR. **A.** Two ISs can lead to the excision of a region of a plasmid by homologous recombination. **B.** This event, which can generate type 1 loss profile (TTSS) and type 2 loss profile (TTSS and upstream locus) in pAsa5, can be assessed by PCR. On this map, the primers (Table 2), shown as arrows, flank one side of their respective IS. In an intact pAsa5 plasmid, these primers are too far apart to generate an amplicon. However, both homologous recombination events involving ISs could place the primers in a suitable position to generate an amplicon. B–C rearrangements can be detected by the 11B1F and 11CR primers, and A–C rearrangements can be detected by the 11AF and 11CR primers. The position of *traC*, *resD* and *exsD* genes are also shown.

To determine whether IS*11*-dependent rearrangements had occurred in the pAsa5 plasmids of the derivative strains displaying a pAsa5 types 1 or 2 loss profiles, appropriate primers had to be designed and the rearranged ISs had to be PCR-amplified. IS-associated primers were tailored to recognize the sequences flanking the corresponding IS (Table 2). However, the pAsa5 primers were not designed to produce amplicons with a standard plasmid configuration. If two identical ISs on the same plasmid undergo homologous recombination, a region of the plasmid might be excised (Figure 2A). The primers were thus designed based on the regions surrounding IS*11*s. A combination of these primers (11B1F and 11CR, or 11AF and 11CR) would thus not produce amplicons under normal circumstances since they were too far apart (Figure 2B). However, if pAsa5 undergoes rearrangements driven by an IS*11*-dependent process, the primers will close up. For example, for a rearrangement involving IS*11*B and IS*11*C (B–C rearrangement), primers 11B1F and 11CR would flank the recombined IS, and would produce an amplicon (Figure 2B) corresponding to type 1 loss profile (loss of the TTSS region). For a rearrangement involving IS*11*A and IS*11*C (A–C rearrangement), primers 11AF and 11CR would produce an amplicon due to the recombination of IS*11*A with IS*11*C.

**Figure 3:**
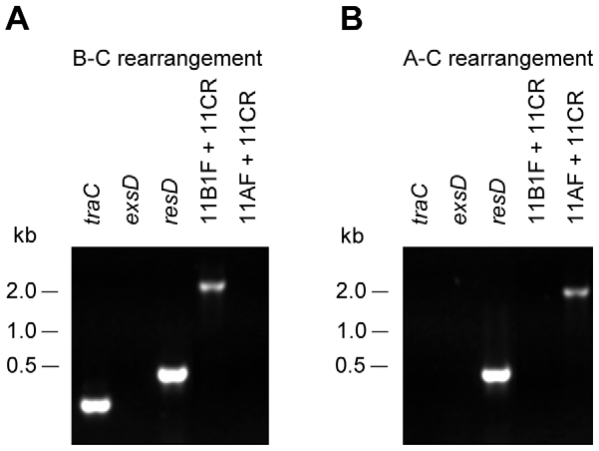
IS*11*-dependent A–C and B–C rearrangements are involved in loss profiles 2 and 1, respectively. A449-R1 derivative strain harboring a loss profile 1 (**A**) and A449-R2 derivative strain harboring a loss profile 2 (**B**) were assessed by PCR, using *exsD*, *traC* and *resD* primers as control (Figure 2B). Primers 11B1F and 11CR were used to assess B–C rearrangement while primers 11AF and 11CR were used to assess A–C rearrangement.

As expected, IS*11*-dependent rearrangements in pAsa5 that led to the loss of TTSS were detected by PCR. Figure 3 shows that B–C and A–C rearrangements were detected by PCR in the A449 derivatives. Control primers targeting the *traC*, *exsD*, and *resD* genes (Figure 2B) were used to confirm the loss profile previously determined by extended genotyping [10] (Figure 3). The *exsD* primers confirmed the presence of the TTSS region, while the *traC* and *resD* primers confirmed the presence of regions upstream and downstream from TTSS, respectively. The loss of PCR signal for *exsD* confirmed type 1 loss profile for A449-R1 (Figure 3A), while the loss of PCR signal for *exsD* and *traC* confirmed type 2 loss profile for A449-R2 (Figure 3B). The B–C rearrangement was associated with type 1 loss profile of the A449-R1 derivative for which a PCR product was generated with 11B1F-11CR primer pair (Figure 3A). The A-C rearrangement was associated with type 2 loss profile of the A449-R2 derivative for which a PCR product was generated with 11AF-11CR primer pair (Figure 3B). These results indicated that the rearrangements observed in pAsa5 involve IS*11*s flanking the lost region.

We attempted to quantify the frequency of IS-dependent rearrangements by testing 26 strains with pAsa5 rearrangements [10] that were derived from the A449 reference strain [11] as well as two Canadian isolates (01-B526 and 01-B516) using the primers described above (Table 2). pAsa5 rearrangements involving an IS-dependent process was detectable using 11B1F-11CR or 11AF-11CR primer pairs in nine out of the 26 derivative strains analyzed (Table 1). In all cases, the 2 kb PCR product obtained was analyzed by amplicon sequencing and BLAST alignment. For each PCR product, the IS*11* was identified with flanking sequences confirming A–C and B–C rearrangements (data not shown).

Interestingly, all the A449 derivative strains and the strains with type 2 loss profile generated a positive PCR signal confirming the involvement of IS*11* in the rearrangement of pAsa5 plasmid in these strains. In the case of type 2 loss profile, strain 01-B526-R4, which had lost the TTSS region as well as a gene 40 kb upstream from the TTSS [10], underwent an A–C rearrangement (Table 1). This unusual loss profile may have resulted from a failed attempt to mobilize the excised genes following the rearrangement involving IS*11*A and IS*11*C. Since all the type 2 loss profiles were associated with IS*11*-dependent rearrangements, this eliminates the involvement of IS*4*, another IS flanking the region corresponding to type 2 loss profile (Figure 1).

Only six out of the 23 derivative strains displaying a type 1 loss profile were associated with a B–C rearrangement through PCR analysis (Table 1). Type 1 loss profiles that could not be explained by B–C rearrangements may have undergone subsequent rearrangements that removed the 11B1F and 11CR annealing sequences or disrupted the alignment of the 11B1F and 11CR primers. This would create a false negative PCR result for IS*11*-dependent rearrangements for the derivative strains displaying type 1 loss profile even if a B–C rearrangement was the initial step of the rearrangement process. These subsequent unpredictable rearrangements could explain the difference between the B–C and A–C rearrangement rates as well as the higher proportion of IS-dependent pAsa5 rearrangement identified for A449 compared to the Canadian isolates. A–C rearrangements may thus lead in the inactivation of the remaining IS*11* in pAsa5, while B–C rearrangements may have no impact on the function of the resulting IS*11*, making subsequent rearrangements with another IS*11* possible. This could explain why type 1 loss profile did not always generate an amplicon with the 11B1F and 11CR primers. For example, a successful IS rearrangement followed by another event that would have disrupted the newly formed IS*11*B/C might have occurred in the 01-B526 and 01-B516 derivatives but not in the A449 derivatives strains, which would explain the failure to detect B–C rearrangements in many of these strains. The presence of other active IS*11*s may lead to these subsequent rearrangements that disrupt the alignment of the 11B1F and 11CR primers.

In addition to the three copies of IS*11* in pAsa5, a copy of IS*11* is present on the chromosome (IS*11*D) and another on pAsal1 (IS*11*E) [11], [12]. It is important to note that 01-B526 and 01-B516 harbor the pAsal1 plasmid while A449 does not [11], [23]. The involvement of these two other IS*11*s, which are located outside pAsa5, was tested in the 01-B526 and 01-B516 derivatives in an attempt to determine why strains with type 1 loss profile do not generate amplicons with the 11B1F and 11CR primers.

**Figure 4:**
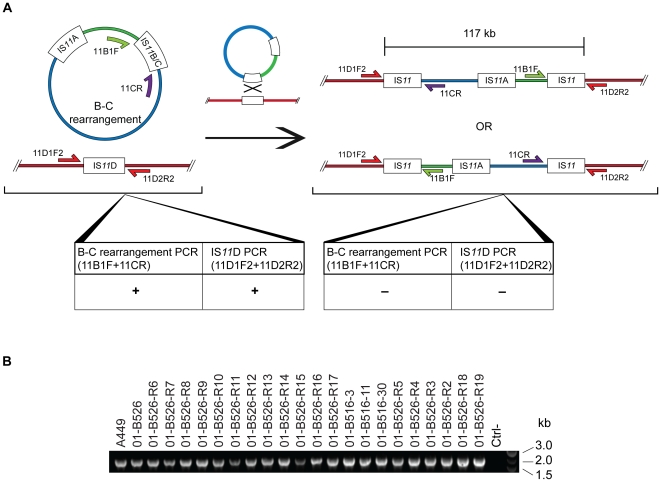
IS*11*D on the chromosome is not involved in pAsa5 insertion sequence rearrangements. Unexplained rearrangements in derivative strains were assessed by PCR using the 11D1F2 and 11D2R2 primers for the regions flanking IS*11*D. **A.** The positions of these primers are illustrated in both scenarios (intact IS*11*D or following homologous recombination in a B–C rearranged pAsa5 plasmid). **B.** An agarose gel electrophoresis of the PCR products, showing a positive result for all tested strains.

For IS*11*D, which is located on the chromosome, the 11D1F2 and 11D2R2 primers flanking IS*11*D were used for the PCR test. If pAsa5 is inserted in the chromosome via IS*11*D, the distance between 11D1F2 and 11D2R2 should increase from 2 kb to over 117 kb, preventing the formation of the PCR product (Figure 4A). The insertion of pAsa5 in the chromosome via IS*11*D was assessed by PCR using the 11D1F2 and 11D2R2 primers for all strains with an unexplained loss profile. Figure 4B shows that a 2-kb amplicon was generated in all the derivative strains, indicating that the chromosomal IS*11*D was intact in all the strains and that it had not participated in the rearrangement of pAsa5.

We intended to assess the involvement of IS*11*E from pAsal1 in the rearrangement of pAsa5 by combining primers for the regions flanking IS*11*E with the 11B1F and 11CR primers. Positive results would have indicated that the sequence had maintained its continuity and that pAsal1 had been inserted into a B–C rearranged pAsa5. However, the structure of pAsal1 made it difficult to create specific primers since pAsal1 shares a 3.8-kb high identity sequence with pAsa3, another *A. salmonicida* plasmid, upstream from IS*11*E. The only major differences between pAsal1 and pAsa3 are that pAsa11 contains the *aopP* TTSS gene and IS*11*E. The similarity between pAsal1 and pAsa3 not only complicates the design of primers for these two plasmids, it can also lead to the sequencing difficulties reported in previous studies [12], [23]. We designed 19 different primer combinations for this PCR approach, none of which were successful since they all generated PCR amplicons in the negative controls (A449 and 01-B526). We also performed long-range PCR using 11B1F and 11CR with primers to detect genes upstream from IS*11*B and downstream from IS*11*C to detect possible insertions of pAsal1 in IS*11*B/C. However, it appeared that the 11B1F and 11CR primers were not specific enough for long-range PCR since they generated large non-specific amplicons in the negative controls (data not shown). This was likely due to the 11B1F primer targeting a 5-kb region that is repeated in pAsa5, while 11CR targets IS*5*, which is located directly downstream from IS*11*C and is also repeated many times in the *A. salmonicida* genome [11] (Figure 1). It was thus not possible to unequivocally assess the recombination of the IS*11*E of pAsal1 with the IS*11*B/C of pAsa5 using a number of technical approaches due to the complex and repeated nature of the *A. salmonicida* genome structure.

Unexplained rearrangements in the 01-B516 and 01-B526 derivatives might result from alternative IS-rearrangements that also excise flanking regions. Alternative rearrangements could explain the difference observed in the fraction of type 1 loss profile that generated a positive B–C rearrangement PCR signal with the A449 and Canadian isolates. The irregular loss profile of 01-B526-R4 was likely due to an irregular IS rearrangement. While the loss profile of 01-B526-R4 closely matched that of type 1 loss profile strains, 01-B526-R4 also lost a portion of the plasmid 40 kb upstream from IS*11*B [10]. Since it undergoes an A–C rearrangement, part of the excised plasmid may have been inserted in another part of the genome. In addition, the B–C rearrangements in the Canadian isolates might have been affected by a defective homologous recombination process that excised the regions flanking IS*11*, deleting portions of the plasmid that were required for the PCR amplification using our primers. Deletions of contiguous DNA by erroneous recombination involving IS*256*-family members have been described for *Enterococcus faecalis* [24] and *Desulfitobacterium* [25]. In both cases, the deletions were detected after exposing the bacterial strains to stressful conditions.

**Figure 5:**
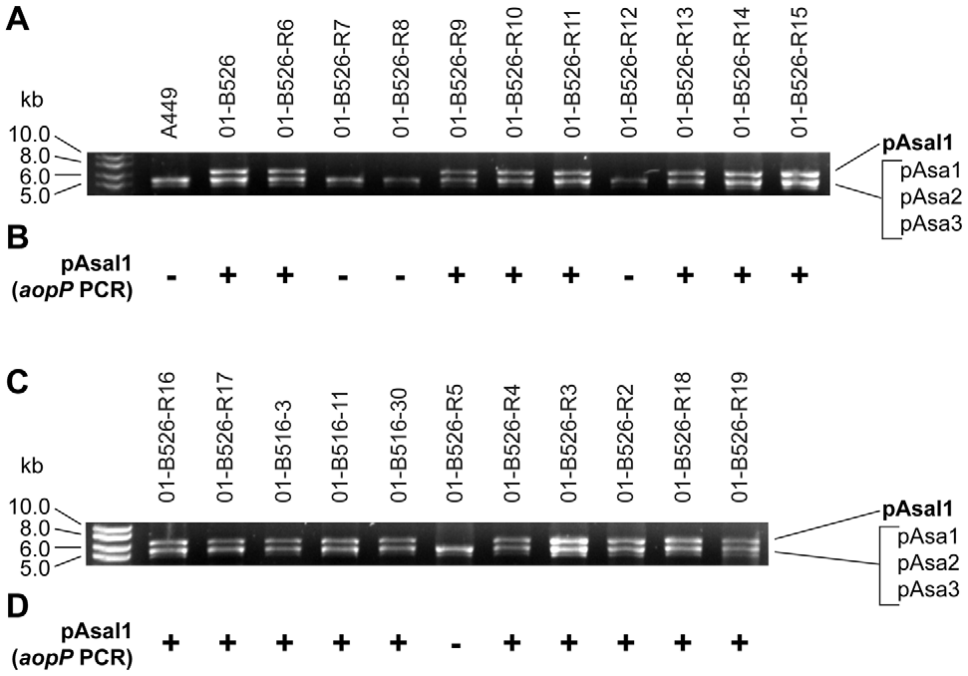
Growing *A. salmonicida* in stressful conditions can lead to the loss of pAsal1. **A and C.** Small plasmids (pAsa1, pAsa2, pAsa3 and pAsal1) from 01-B526 and 01-B516 derived strains were purified and digested using EcoRI leading to the linearization of the plasmids [12]. An agarose gel electrophoresis of the digested plasmids is shown. **B and D.** DNA lysates of the same strains were tested by PCR with pAsal1 primers (Table 2) to confirm by a second approach the presence or the absence of pAsal1 in these strains. The presence (+) or the absence (−) of a PCR product is indicated.

Stressful growth conditions have a deleterious effect on the integrity of pAsa5 due to the presence of IS*11*s in the plasmid. We thus determined whether pAsal1 was also affected by stressful growth conditions given that it also bears an IS*11*. We verified the presence of pAsal1 in the 01-B526 and 01-B516 derivatives using two approaches. First, plasmid DNA from the 01-B526 and 01-B516 derivatives was purified, digested with EcoRI, and separated by agarose gel electrophoresis. This method is commonly used to detect pAsal1, which migrates as a single band above the other small plasmids usually found in *A. salmonicida* [12]. Figure 5A and C shows that four derivatives had lost the 6-kb band of pAsal1. To confirm the loss of pAsal1, a PCR test using specific pAsal1F and pAsal1R primers that target the *aopP* gene specific to pAsal1 [12], [14] (Table 2) showed an absence of signal for the same four derivative strains (Figure 5B and D) than those displaying an absence of pAsal1 by plasmid digestion analysis. This suggested that pAsal1 is an unstable plasmid and may help to explain why it was not observed in all the strains assessed by other investigators [12], [26].

### Conclusion

In the present study, we attempted to determine whether the heat-induced pAsa5 rearrangements observed in *A. salmonicida* are driven by IS*11*. The mobilization of IS*11* can explain both types 1 and 2 loss profiles in reference strain A449 that were generated by B–C and A–C rearrangements, respectively confirming that IS-dependent rearrangements occur in *A. salmonicida* and that these mobile DNA elements are involved in some events leading to the loss of the TTSS and of virulence [10]. Our results also showed that these rearrangements can be triggered by stressful conditions such as heat, although this cannot be detected by PCR in an optimal manner. Thus, for the 01-B526 and 01-B516 strains the loss profiles could not always be explained using the IS-rearrangement PCR method. It is possible that IS-rearrangement homologous recombination in these strains might be defective or might involve other rearrangement events, which would make it impossible to classify them as B–C rearrangements using the IS-rearrangement PCR approach, even if IS*11*B and IS*11*C played a role in the loss of the TTSS region. Such defective rearrangements might have also generated the unique 01-B526-R4 loss profile following an A–C rearrangement event. An additional plasmid pAsal1 in the Canadian isolates might also have been involved in IS-dependent rearrangements, but high identity repeated regions flanking some IS*11*s in *A. salmonicida* prevented the design of the highly specific primers required to answer this question. Despite these limits, it is interesting to note that this is the first study on the mechanistic aspects of IS-dependent rearrangements in *A. salmonicida*. Since the *A. salmonicida* genome contains a high number of ISs [11], it will be interesting to assess the plasticity of the *A. salmonicida* genome that is associated with ISs and to determine whether stressful conditions other than growth at 25°C can promote pAsa5 rearrangements.
